# Supplementary material for: A Deep Learning Model Integrating Clinical and MRI Features Improves Risk Stratification and Reduces Unnecessary Biopsies in Men with Suspected Prostate Cancer
Source: Cancers (Basel). 2025 Jul 7;17(13):2257. doi: 10.3390/cancers17132257 (PMC12248439; doi:10.3390/cancers17132257)
Supplement: Supplementary file 1 [file cancers-17-02257-s001.zip › cancers-3695800-supplementary.pdf]

Supplementary Figure S1a. Calibration plot for Model 1 shows a tendency to underpredict clinically significant prostate cancer.

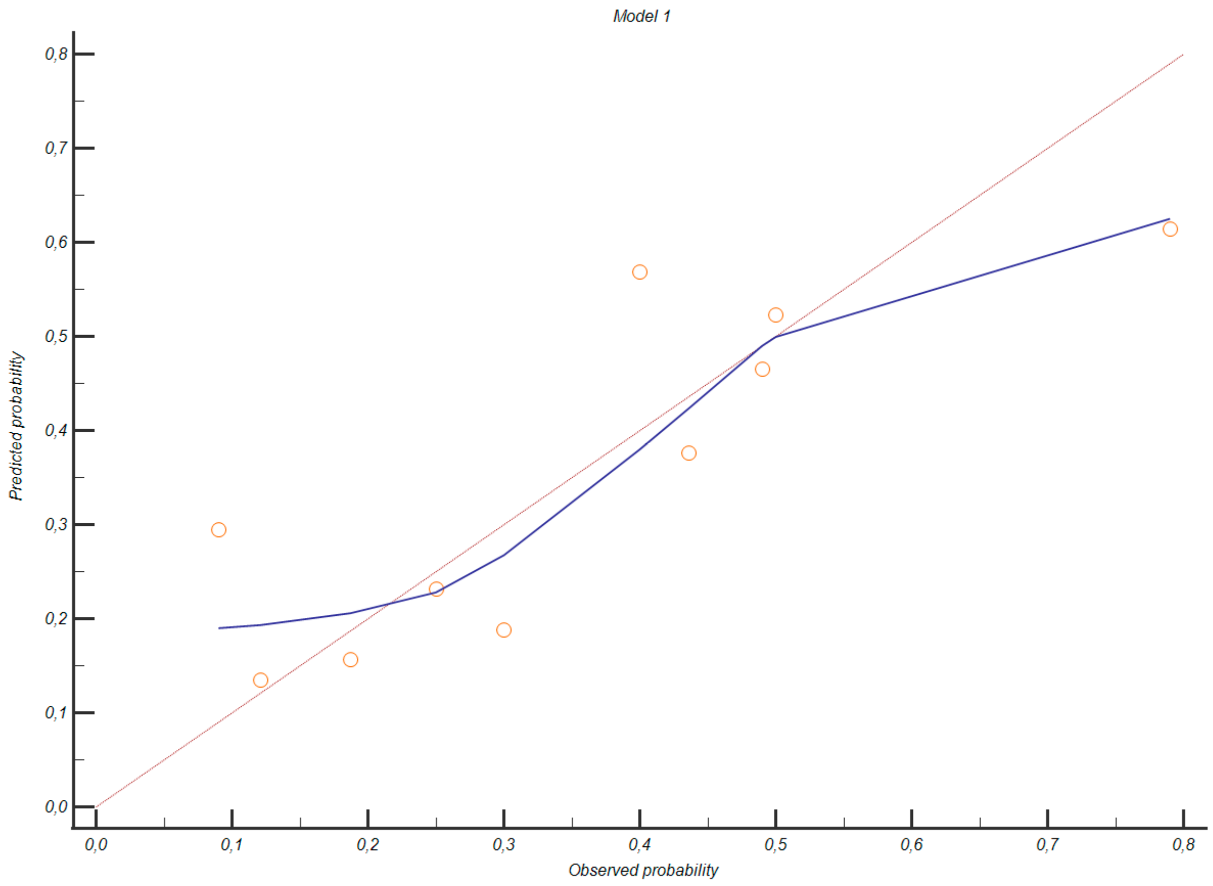

Supplementary Figure S1b. Calibration plot for Model 2 shows a tendency to underpredict clinically significant prostate cancer for PI-RADS categories 1 and 2, and 5, while overpredicting for PI-RADS categories 3 and 4.

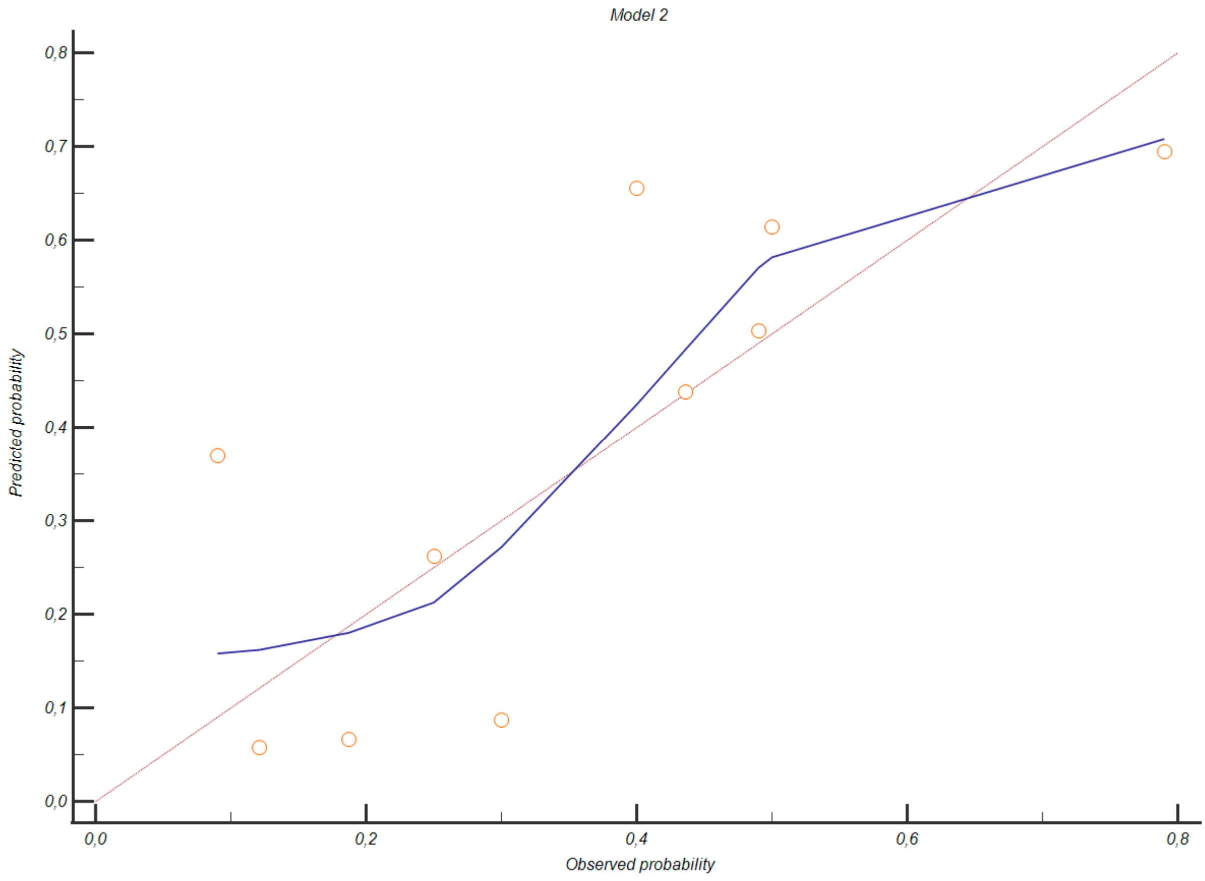

Supplementary Figure S1c. Calibration plot for Model 3 shows a closer alignment between predicted and observed probabilities of clinically significant prostate cancer along the diagonal reference line, albeit with a slight overall tendency toward overprediction.

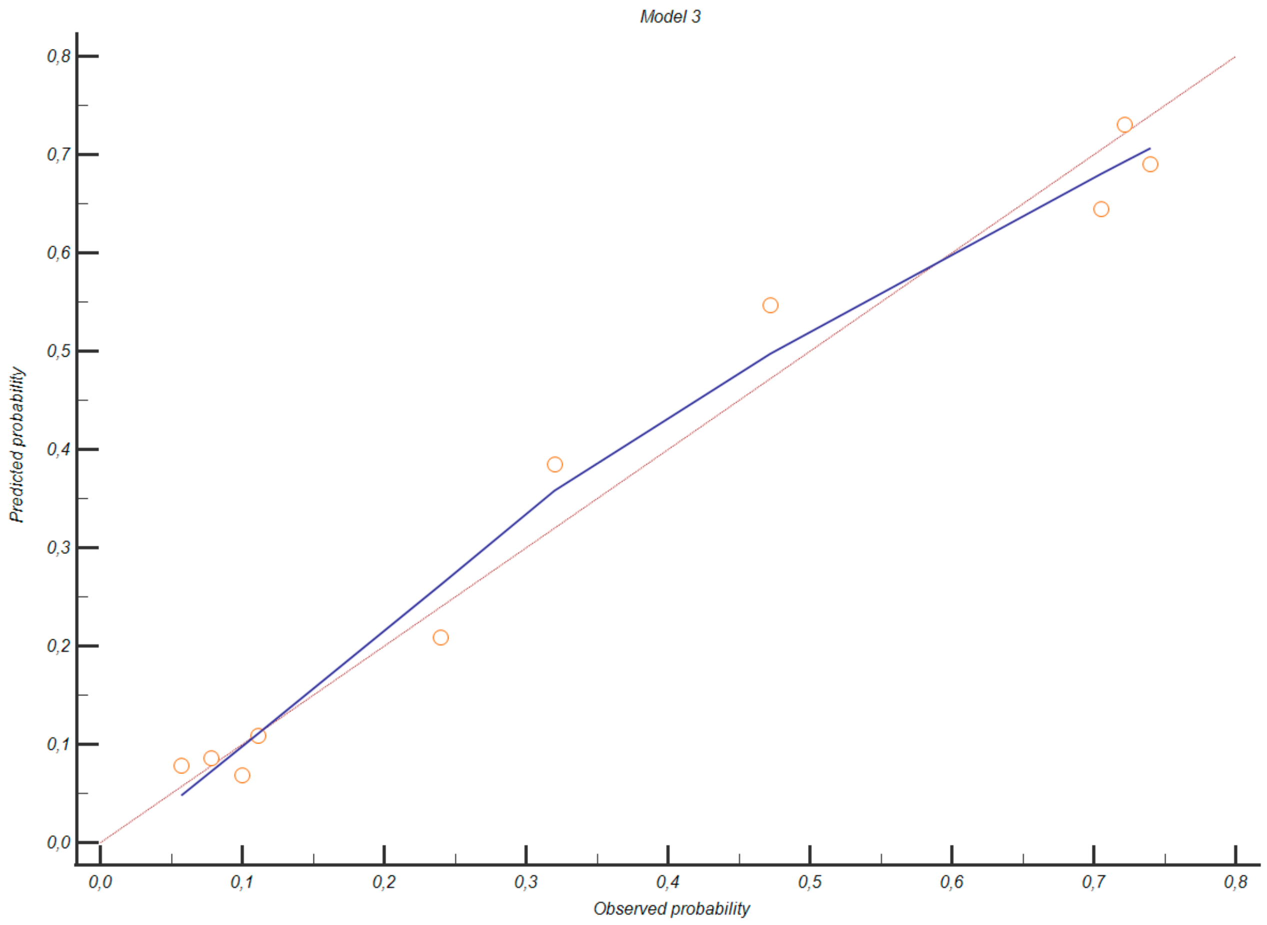

	Double sequence DWI		T2-weighted imaging	DCE
	First sequence*	Second sequence**		
**Sequence design**	SS-EPI	SS-EPI	TSE	THRIVE
**Weighting**	DWI	DWI	T2	T1
**Acquisition plane**	Transverse	Transverse	Transverse/coronal/sagittal (at least two planes)	Transverse
**TR (ms)**	5350	5424	4727/3076/3714	3.5
**TE (ms)**	68	78	80/80/80	1.77
**Echo train length**	-	-	8/16/16	-
**EPI factor**	109	109	-	-
**Half scan factor**	0.62	0.62	No	no
**FOV (mm x mm)**	200 x 200	200 x 200	180x180/180x180/180x180	200x200
**Acquisition voxel size**	2 x 2 x 3	2 x 2 x 3	0.6 x 0.6 x 3	1.2 x 1.2 x 8
**Reconstruction pixel size (mm x mm x mm)**	1.4 x 1.4 x 3	1.4 x 1.4 x 3	0.45 x 0.45 x 3	0.63 x 0.63 x 4
**Number of slices**	24	24	24/20/20	20
**Interslice gap (mm)**	0	0	0/0/0	0
**b-values (s/mm^2)/number of excitations**	100/1, 500/1, 1000/2	100/1, 2000/3	-	-
**Number of excitations**	-	-	2/1/1	1
**Fat saturation**	SPAIR	SPAIR	-	Spectral fat saturation
**Parallel imaging (x acceleration factor)**	SENSE x 2	SENSE x 2	SENSE x 2.5/1/1	SENSE x 2
**Acquisition time (min)**	3.2	3.2	5.3/4.2/5.1	Total acquisition time 6 (0.14 x 44 acquisitions)

* Used to build the apparent diffusion coefficient map by fitting signal intensity versus the b-values up to 1000 s/mm^2.

** Used to provide the maximum b-value images (b = 2000 s/mm^2).

Supplementary Table S1. Acquisition parameters for 3.0 Tesla MRI of the prostate.

DWI = diffusion-weighted imaging; TSE-T2WI = turbo spin echo T2-weighted imaging; DCE = dynamic contrast-enhanced; THRIVE = T1-weighted high-resolution isotropic volume examination; SS-EPI = single-shot echo-planar imaging; TR = time of repetition; EPI = echo-planar imaging; TE = time of echo; FOV = field of view; SENSE = sensitivity encoding; SPAIR = spectral adiabatic inversion recovery

	Double sequence DWI		T2-weighted imaging	DCE
	First sequence*	Second sequence**		
**Sequence design**	SS-EPI	SS-EPI	TSE	VIBE
**Weighting**	DWI	DWI	T2	T1
**Acquisition plane**	Transverse	Transverse	Transverse/coronal/sagittal (at least two planes)	Transverse
**TR (ms)**	4100	4100	2810/2810/2900	4.5
**TE (ms)**	64	68	105/105/113	1.66
**Echo train length**	-	-	8/16/16	-
**EPI factor**	122	122	-	-
**Half scan factor**	0.75	0.75	No	No
**FOV (mm x mm)**	200x200	200x200	200x200	280x280
**Acquisition voxel size**	1.6x1.6x3.5	1.6x1.6x3.5	0.7 x 0.5 x 3	1.68 x 1.09 x 5
**Reconstruction pixel size (mm x mm x mm)**	Same as above	Same as above	0.5 x 0.5 x 3	1.1 x 1.1 x 3
**Number of slices**	20	20	35/35/35	30
**Interslice gap (mm)**	0	0	0/0/0	-
**b-values (s/mm^2)/number of excitations**	100/2, 600/7, 1000/12	100/4, 1200/12	-	-
**Number of excitations**	-	-	3	1
**Fat saturation**	SPAIR	SPAIR	-	Spectral
**Parallel imaging (x acceleration factor)**	SENSE (2)	SENSE (2)	SENSE (2)	SENSE (2)
**Acquisition time (min)**	4.4	3.4	5.1/5.1/5.2	3.25

* Used to build the apparent diffusion coefficient map by fitting signal intensity versus the b-values up to 1000 s/mm^2.

** Used to provide the maximum b-value images (b = 1400 s/mm^2, obtained by interpolation).

Supplementary Table S2. Acquisition parameters for 1.5 Tesla magnetic resonance imaging (MRI) of the prostate.

DCE = dynamic contrast-enhanced; DWI = diffusion-weighted imaging; FOV = field of view; SENSE = sensitivity encoding; SPAIR = spectral adiabatic inversion recovery; TE = time of echo; TR = time of repetition; TSE = turbo spin echo; SS-EPI = single-shot echo-planar imaging; VIBE = volumetric interpolated breath-hold examination; EPI = echo planar imaging.
